# Vitamin D status and association with gestational diabetes mellitus in a pregnant cohort in Iceland

**DOI:** 10.29219/fnr.v65.5574

**Published:** 2021-03-23

**Authors:** Kristin S. Magnusdottir, Ellen A. Tryggvadottir, Ola K. Magnusdottir, Laufey Hrolfsdottir, Thorhallur I. Halldorsson, Bryndis E. Birgisdottir, Ingibjorg T. Hreidarsdottir, Hildur Hardardottir, Ingibjorg Gunnarsdottir

**Affiliations:** 1 Unit for Nutrition Research, Landspitali University Hospital and Faculty of Food Science and Nutrition, University of Iceland, Reykjavik, Iceland; 2 Institution of Health Science Research, University of Akureyri and Akureyri Hospital, Akureyri, Iceland; 3 Centre for Fetal Programming, Department of Epidemiology Research, Statens Serum Institut, Copenhagen, Denmark; 4 Department of Obstetrics and Gynecology, Landspitali University Hospital, Reykjavík, Iceland; 5 Faculty of Medicine, University of Iceland, Reykjavík, Iceland

**Keywords:** vitamin D, pregnancy, supplements, gestational diabetes mellitus, nutritional status, cod liver oil

## Abstract

**Background:**

Vitamin D deficiency has been associated with an increased risk of gestational diabetes mellitus (GDM), one of the most common pregnancy complications. The vitamin D status has never previously been studied in pregnant women in Iceland.

**Objective:**

The aim of this research study was to evaluate the vitamin D status of an Icelandic cohort of pregnant women and the association between the vitamin D status and the GDM incidence.

**Design:**

Subjects included pregnant women (*n* = 938) who attended their first ultrasound appointment, during gestational weeks 11–14, between October 2017 and March 2018. The use of supplements containing vitamin D over the previous 3 months, height, pre-pregnancy weight, and social status were assessed using a questionnaire, and blood samples were drawn for analyzing the serum 25‑hydroxyvitamin D (25OHD) concentration. Information regarding the incidence of GDM later in pregnancy was collected from medical records.

**Results:**

The mean ± standard deviation of the serum 25OHD (S-25OHD) concentration in this cohort was 63±24 nmol/L. The proportion of women with an S-25OHD concentration of ≥ 50 nmol/L (which is considered adequate) was 70%, whereas 25% had concentrations between 30 and 49.9 nmol/L (insufficient) and 5% had concentrations < 30 nmol/L (deficient). The majority of women (*n* = 766, 82%) used supplements containing vitamin D on a daily basis. A gradual decrease in the proportion of women diagnosed with GDM was reported with increasing S-25OHD concentrations, going from 17.8% in the group with S-25OHD concentrations < 30 nmol/L to 12.8% in the group with S-25OHD concentrations ≥75 nmol/L; however, the association was not significant (*P for trend* = 0.11).

**Conclusion:**

Approximately one-third of this cohort had S-25OHD concentrations below adequate levels (< 50 nmol/L) during the first trimester of pregnancy, which may suggest that necessary action must be taken to increase their vitamin D levels. No clear association was observed between the vitamin D status and GDM in this study.

## Popular scientific summary

- Vitamin D supplementation is encouraged by the Icelandic authorities and was quite widespread (80%) in the population studied.
- Approximately one-third of this cohort reported S-25OHD concentrations below adequate levels (< 50 nmol/L) during the first trimester of pregnancy.
- No clear association was seen between vitamin D status and GDM in the population studied.

Vitamin D deficiency or insufficiency during pregnancy is believed to be a common problem reported worldwide (1). However, the vitamin D status of pregnant women has never been studied in Iceland. In countries that receive limited sunshine, such as Iceland, vitamin D formation in the skin can be very limited, especially during the winter months (from October to March) (2, 3). A very few natural sources of vitamin D exist, and all Icelanders are, therefore, encouraged to consume vitamin D in the form of supplements or cod liver oil, especially during the winter season (4).

Although the most well-established role of vitamin D involves the control of blood calcium concentrations and the maintenance of healthy bones (5), vitamin D deficiency has also been associated with several other conditions and diseases, including gestational diabetes mellitus (GDM) (1, 6). The mechanisms that underlie this association are not fully understood and are thought to be manifold (7). Vitamin D receptors are expressed by many different cells, including muscle cells and pancreatic beta cells (8), and may influence glucose metabolism, insulin secretion, and insulin resistance (9, 10). Studies that have investigated the association between the vitamin D status and the increased gestational diabetes risk have been somewhat conflicting, with some studies showing that vitamin D is associated with an increased risk of GDM, and others reporting no relationship between these two factors (11).

The primary aim of this study was to assess the vitamin D status of pregnant women in Iceland and to determine whether the intake of dietary supplements containing vitamin D was associated with 25‑hydroxyvitamin D (25OHD) concentrations in the serum. The secondary aim of this study was to investigate the association between the vitamin D status and the gestational diabetes incidence.

## Methods

### Subjects

All women who visited the Prenatal Diagnostic Unit at Landspitali National University Hospital, Reykjavik, Iceland during gestational weeks 11–14, between 2 October 2017 and 28 March 2018, were invited to participate in this study. During the study period (6 months), 1,684 women were scheduled to undergo their first ultrasound screening at Landspitali, corresponding to approximately 77% of the total pregnant population in Iceland. Of these 1,684 women, 244 women (15%) were excluded from the study because they did not speak Icelandic and could, therefore, not respond to the questionnaire. Other exclusion criteria included women outside of the gestational weeks 11–14 defined in this study, women who failed to appear at their scheduled appointment times, and women who experienced miscarriage, which resulted in the exclusion of an additional 90 women. Of the remaining 1,350 women deemed to be eligible for participation, 329 declined to participate for various reasons. Of the 1,015 women who were enrolled in this study, blood samples were obtained from 942 women. Information regarding GDM diagnosis later in pregnancy was retrieved from the medical records of 837 women who also had their blood drawn for the assessment of vitamin D status. The study was approved by the National Bioethics Committee and the Medical Directorate of Landspitali University Hospital. Written informed consent was obtained from all participants.

### Assessment of vitamin D status

Blood samples were obtained from subjects during gestational weeks 11–14. Serum samples were stored at −80°C until analysis of serum 25OHD (S-25OHD) concentration using an electrochemiluminescence immunoassay at the Clinical Core Laboratory, Landspitali University Hospital, which was performed during spring 2019. Control samples, which are measured daily by the laboratory, have shown that the CV% of this analysis method is approximately 4–5%. S-25OHD concentrations ≥ 50 nmol/L were considered adequate, 30–49.9 nmol/L were considered insufficient, and < 30 nmol/L was defined as deficient (2, 5). We also report the number and rate of women with S-25OHD concentrations ≥ 75 nmol/L, as no consensus exists regarding optimal S-25OHD levels, and some researchers use a cut-off value of 75 nmol/L as an indicator of adequate vitamin D status (12, 13).

### Dietary and supplement intake and background variables

Women who agreed to participate in this study answered a questionnaire presented in an electronic format. The questionnaire included questions regarding background information, including maternal age, education, smoking habits, parity, nausea in pregnancy, pre-pregnancy weight, and height. Information regarding weight and height was used to calculate the pre-pregnancy body mass index (BMI, in kg/m^2^). BMI < 18.5 kg/m^2^ was defined as underweight, 18.5–24.9 kg/m^2^ as normal weight, 25–29.9 kg/m^2^ as overweight, and ≥ 30.0 kg/m^2^ as obese.

Supplement intake during the previous 3 months (starting at approximately the onset of pregnancy) was assessed by a short food frequency questionnaire (FFQ), which included questions regarding the frequency of cod liver oil (a traditional source of vitamin D in Iceland), vitamin D supplement, and multivitamin consumption. Information regarding the consumption of other potential dietary sources of vitamin D, such as vitamin Denriched milk and oily fish, was also assessed through the FFQ. The development of the FFQ has previously been described in detail (14–16).

### GDM

Information regarding the occurrence of GDM was retrieved from maternal hospital records (ICD-10 codes O24.4 and O24.9, but O24.9 is used at Landspitali GDM treated with medications). The criteria for GDM diagnoses were based on the recommendations of the 2010 International Association of Diabetes and Pregnancy Study Groups (IADPSG) Consensus Panel (17). Other information gathered from medical records included gestational age, family history of diabetes mellitus, and measured weight at the first and last maternal care visits, and were used to calculate the total weight gain during pregnancy.

## Statistical analysis

Data from the dietary questionnaire and maternal hospital records were entered in SPSS (IBM SPSS Statistics, version 26), where all statistical analysis was conducted. Data are expressed as mean ± standard deviation (SD) for normally distributed variables and as the median and interquartile range (IQR) for skewed variables. Dichotomous variables are reported as frequencies and percentages (%). For continuous variables, an independent sample T-test was used to formally test the significance of differences between the two groups of normally distributed variables, and the Mann-Whitney U test was used to assess the differences among skewed variables. The Chi-square test was used to test differences in dichotomous variables across groups.

Logistic regression analysis was used to examine associations between categories of S-25OHD status (< 30, 30–49.9, 50–74.9, and ≥ 75 nmol/L) and GDM occurrence. The results are presented as odds ratios (ORs) with 95% confidence intervals (95% CIs), both before and after adjustment for covariates, using the deficient S-25OHD status (< 30 nmol/L) as the reference category. The covariates included in our adjusted models were maternal age, pre-pregnancy BMI, parity, and smoking during pregnancy. As a formal test for an association, we used the Chi-square test by modeling categorical variables as continuous terms in the regression model and using the median S-25OHD value for each category. *P* < 0.05 was considered to be statistically significant. All reported *P*-values are two-sided.

## Results

The characteristics of the study participants are presented in Table 1, both for the whole cohort and for subgroups of women with and without a later GDM diagnosis. Maternal age was 25–34 years for 66% of cases, and the median (IQR) pre-pregnancy BMI was 24.4 (6.3) kg/m^2^. Approximately 93% of participants were married or lived with a partner. Women who later developed GDM were more likely to be primi/multiparous and had higher pre-pregnancy BMI [median (IQR) 26.6 (8.8) kg/m^2^ vs. 24.2 (5.9) kg/m^2^] but lower gestational weight gain [mean (SD) 9.7 (6.1) kg vs. 12.7 (5.2) kg] than those who did not develop GDM. No association was found between the vitamin D status and BMI or social status, other than marital status. Single mothers tended to have lower S-25OHD concentrations than married or partnered mothers (*P* < 0.05).

**Table 1:** Characteristics of the subjects divided according to the diagnosis of gestational diabetes

Characteristics	All (*n* = 938)		GDM (*n* = 126)		Non-GDM (*n* = 711)		P^a^
Maternal age (year), *n* (%)	*n*	%	*n*	%	*n*	%	0.06^b^
18–24	144	15.3	14	11.1	114	16.0	
25–29	341	36.2	48	38.1	261	36.7	
30–34	281	29.8	33	26.2	218	30.7	
35–39	138	14.6	24	19.0	95	13.4	
40–45	27	2.9	7	5.6	19	2.7	
Parity, *n* (%)							0.03^b^
Nulliparous	411	43.6	51	40.5	316	44.4	
Primi/multiparous	524	55.6	73	57.9	394	55.4	
Marital status, *n* (%)							0.76^b^
Married/cohabitant	874	92.7	106	95.1	661	95.0	
Single	42	4.5	6	4.9	35	5.0	
Smoking in pregnancy, *n* (%)	43	4.6	7	5.6	31	4.4	0.12^b^
Education level, *n* (%)							0.34^b^
Less than elementary school	5	0.5	0	0.0	5	0.7	
Elementary school	104	11.0	16	12.9	73	10.3	
High school and technical school	273	29.0	38	30.6	208	29.3	
Bachelor’s degree	317	24.9	35	28.2	257	36.2	
Master’s or doctorate degree	235	24.9	35	28.2	167	23.5	
Height (cm), mean ± SD	167.5 ± 7.4		168.1 ± 5.2		167.3 ± 7.8		0.34^c^
Pre-pregnancy weight (kg), mean ± SD	72.3 ± 15.7		79.2 ± 19.0		71.3 ± 15.0		<0.01^d^
Pre-pregnancy body mass index (BMI), median (IQR)	24.4	6.3	26.5	8.8	24.2	5.9	<0.01^d^
Pre-pregnancy BMI (groups), *n* (%)							<0.01^b^
<18.5 kg/m^2^	18	1.9	2	1.6	13	1.8	
18.5–24.99 kg/m^2^	498	52.9	50	41.0	388	55.0	
25–29.99 kg/m^2^	240	25.5	30	24.6	186	26.4	
≥30 kg/m^2^	171	18.2	40	32.8	118	16.7	
Gestational weight gain (kg), mean ± SD	12.3 ± 6.9		9.7 ± 6.1		12.7 ± 5.2		<0.01^c^
Gestational week at delivery, median (interquartile range)	39.7	3.3	39.6	2.3	39.9	2.7	0.76^d^
Family history of type 2 diabetes, *n* (%)	11	1.2	2	1.8	6	1.0	<0.01^b^

Missing data in the total group: maternal age *n* = 7; height *n* = 7; pre-pregnancy weight *n* = 11; pre-pregnancy BMI *n* = 11; pre-pregnancy BMI (groups) *n* = 11; parity *n* = 3; marital status *n* = 22; prenatal smoking *n* = 8; education level *n* = 4. Information on GDM diagnosis was available for 837 subjects.

a Differences between GDM and non-GDM.

b Chi-square test for differences among groups.

c T-test for differences among groups.

d Mann-Whitney U test for difference among groups.

Table 2 shows the vitamin D status of the whole cohort, as well as the use of vitamin D supplements. The proportion of women with S-25OHD concentrations ≥ 50 nmol/L (adequate) was 70%, whereas 25% of them had S-25OHD concentrations of 30–49.9 nmol/L (insufficient) and 5% had concentrations < 30 nmol/L (deficient). The majority of women (*n* = 766, 82%) who consumed supplements of vitamin D daily had a mean ± SD S-25OHD concentration of 66 ± 24 nmol/L. Interestingly, approximately 24% of women who took vitamin D supplements daily were classified as having an insufficient vitamin D level (< 50 nmol/L). Women who reported never using vitamin D supplements (*n* = 104, 11%) had a mean ± SD S-25OHD concentration of 45 ± 18 nmol/L. In this group, approximately 66% of women were defined as having an insufficient S-25OHD concentration (< 50 nmol/L), including 18% who were defined as deficient (< 30 nmol/L). Both non-users and irregular users of vitamin D supplements had significantly reduced S-25OHD concentrations compared with those taking daily supplements containing vitamin D (*P* < 0.01). A majority of the women (89%) who had sufficient vitamin D status (≥ 50 nmol/L) used vitamin D supplements on a daily basis.

**Table 2:** S-25OHD concentration (nmol/L) in all subjects (*n* = 938) and according to the use of supplements containing vitamin D (*n* = 935)

	*n*	mean ± SD	<30 nmol/L		30–49.9 nmol/L		50–74.9 nmol/L		≥ 75 nmol/L	
			*n*	%	*n*	%	*n*	%	*n*	%
All subjects	938	63.0 ± 24.4	51	5.4	234	24.9	398	42.4	255	27.2
Not taking any supplements containing vitamin D	104	44.6 ± 17.5	19	18.3	50	48.1	29	27.9	6	5.8
Irregular use of supplements containing vitamin D*	65	55.1 ± 21.1	6	9.2	24	36.9	25	38.5	10	15.4
Daily vitamin D supplementation	766	65.9 ± 24.1	26	3.4	160	20.9	342	44.6	238	31.1

* Subjects reporting use of vitamin D supplements from 1–2 times per month up to 4–6 times per week.

The amounts of vitamin D found in the most commonly used supplements available in Icelandic markets are shown in Supplementary file. The number (%) of subjects taking various daily supplements containing vitamin D, their 25OHD concentrations, and the number (%) of subjects categorized as having deficient (< 30 nmol/L), insufficient (< 50 nmol/L), and sufficient (≥ 50 nmol/L) vitamin D status are also shown in the same Supplementary file. Relatively few women used vitamin D (1 μg/100 g) enriched milk daily (*n* = 100). A large majority of women who consumed vitamin D-enriched milk daily also consumed daily supplements containing vitamin D (*n* = 81). The average frequency of oily fish consumption was < 0.5 times per week. The consumption of neither vitamin D-enriched milk nor oily fish was associated with vitamin D status.

Medical records were obtained for 837 women, including 126 women (15%), who were eventually diagnosed with GDM (Table 3). The mean ± SD S-25OHD concentration of the GDM group was 60 ± 24 nmol/L, compared with 63 ± 24 nmol/L in the non-GDM group (*P* > 0.05). Approximately 35% of subjects with GDM and 30% of non-GDM subjects had S-25OHD concentrations < 50 nmol/L. A gradual decrease in the proportion of women diagnosed with GDM was found to occur with increasing S-25OHD concentrations, going from 17.8% in the group with S-25OHD concentrations < 30 nmol/L to 12.8% in the group with S-25OHD concentrations ≥ 75 nmol/L (*P* for trend = 0.17). After adjustment for covariates, as presented in Table 3, the association was somewhat strengthened but remained non-significant (*P* = 0.11). The OR for GDM among women with S-25OHD concentrations ≥ 75 nmol/L compared with those with S-25OHD concentrations < 30 nmol/L was 0.60 (95%CI: 0.25, 1.45).

**Table 3:** Vitamin D status in subjects who later were diagnosed with gestational diabetes mellitus (*n* = 126) or not (total number of subjects included in the analysis *n* = 837)

	Serum 25OHD (nmol/L)				*P* for trend^a^
	<30	30–49.9	50–74.9	≥75	
No cases (%)/*n*	8 (17.8%)/45	36 (17.2%)/209	53 (14.9%)/356	29 (12.8%)/227	
Unadjusted OR (95% CI)	1.00	0.96 (0.41, 2.24)	0.81 (0.36, 1.83)	0.68 (0.29, 1.60)	0.17
Adjusted OR (95% CI)^b^	1.00	0.90 (0.38, 2.12)	0.77 (0.33, 1.76)	0.60 (0.25, 1.45)	0.11

a Chi-square test.

b Adjusted for maternal age, parity and maternal pre-pregnancy body mass index, and smoking during pregnancy.

## Discussion

In this study, 70% of the women (*n* = 942) had S-25OHD concentrations ≥ 50 nmol/L, which is considered adequate. The remaining 30% had insufficient S-25OHD concentrations, with 25% presenting S-25OHD concentrations between 30 and 49.9 nmol/L (insufficient) and 5% presenting S-25OHD concentrations < 30 nmol/L (deficient). Approximately 27% of the women had S-25OHD concentrations ≥ 75 nmol/L. No clear association was observed between the vitamin D status and GDM in this study.

A majority of subjects in this study (approximately 82%) used supplements containing vitamin D daily, in line with guidelines established by Icelandic health authorities (18). The frequency of vitamin D supplement intake in Iceland appears to be higher in this study than has been reported in other countries (19). The most common type of vitamin D supplements used in this study included vitamin D tablets (51%), whereas fewer than 20% of participants reported using the traditional Icelandic source of vitamin D, cod liver oil. A concerning finding was that 25% of those who claimed they used supplements daily had inadequate vitamin D status. Some but not all of these participants reported the use of multivitamin supplements, which typically contain 5–10 μg (200–400 IU) vitamin D in a daily dose. This dose is lower than the current 15 μg/day (600 IU) recommended daily intake (RDI) for adults, including pregnant women, established by Icelandic health authorities (18). In comparison, most single-nutrient vitamin D supplements available on the Icelandic market contain 25–50 μg (1,000–2,000 IU) vitamin D per daily serving. Some women may have only recently begun to take supplements containing vitamin D, and may have entered pregnancy with low S-25OHD concentrations. The RDI for vitamin D is the required amount necessary to maintain an adequate vitamin D status, and may not be high enough to correct an insufficient status (20, 21). Van Groningen et al. (22) developed a practical method for calculating the vitamin D dose required to rapidly correct vitamin D deficiency in individuals using the following equation to estimate the necessary loading dose to increase vitamin D levels to 50 nmol/L: dose (IU) = 40 × [50 − serum 25-OHD (nmol/L)] × [body weight (kg)]. According to this equation, a 72-kg woman with a pre-pregnancy S-25OHD concentration of 24 nmol/L would require 6 months to achieve an S-25OHD concentration of 50 nmol/L when consuming 10 μg vitamin D daily, which is the amount of vitamin D provided in many of the most commonly used multivitamins on the Icelandic market. If the goal is to achieve an S-25OHD concentration > 50 nmol/L before week 20, the dose would need to be increased to as high as 45 μg/day (1,800 IU). In a recent study, a dose of 30 μg/day (1,200 IU) was suggested to be necessary to maintain a sufficient vitamin D status (50 nmol/L) for the majority of pregnant, white-skinned women at northern latitudes, which would also maintain an umbilical cord S-25OHD concentration of ≥ 25–30 nmol/L for almost all newborns (23). Other studies have suggested that doses greater than the current RDI dose are necessary to maintain adequate vitamin D levels in pregnancy (24–26).

Previous studies conducted to examine the association between the vitamin D status and GDM risk have reported somewhat conflicting results. Recent systematic reviews and meta-analyses found a relationship between low vitamin D status (insufficient or deficient) and an increased risk of GDM (11–13, 27–29). These studies also reported that vitamin D levels were lower in women with GDM than in those with normal glucose levels. However, in this study, no significant difference in vitamin D status was observed between women who developed GDM later in pregnancy and those who did not, and no significant relationship was identified between low vitamin D levels and an increased risk of GDM. The proportion of women with vitamin D deficiency (S-25OHD < 30 nmol/L) was relatively low in our cohort, and the number of women taking supplements containing vitamin D may have been higher than in previous studies (these data are not always reported). Clinical trials examining the effect of vitamin D supplements on GDM patients have yielded unclear results (30). Researchers have identified several possible factors that might confound results. For example, low vitamin D concentrations might not be a contributing factor for GDM, or a causal relationship may exist in the opposite direction. In addition, factors associated with study design and whether the subjects were at high risk of developing GDM or vitamin D deficiency at baseline could also affect the outcomes. The supplementation doses, supplementation periods, and methods used to assess S-25OHD concentrations can vary, and measurements may be performed at different pregnancy stages. In some studies, all participants were supplemented with vitamin D for ethical reasons, which could also affect the results (24, 31, 32).

The primary strengths of this study are the inclusion of a large sample size and a high participation rate. According to Statistics Iceland, 2,188 infants were born in Iceland between April 2018 and September 2018 (which corresponds with the expected delivery dates for women attending 11–14-week ultrasound examinations during the study period). This study, therefore, includes 45% of the total population of pregnant women in Iceland. One of the limitations may be that recruitment was limited to the capital of Reykjavik. According to Statistics Iceland, approximately 70% of women in Iceland live in the capital region. However, we cannot exclude the possibility that the vitamin D status of women living outside the capital area may be different from that of the women included in this study. Furthermore, the number of women that were excluded for not speaking Icelandic indicates the importance of including English versions of the questionnaires for future studies performed in Iceland. The vitamin D status of pregnant women in Iceland has never been studied before; therefore, this study provides new and important information regarding the vitamin D status of this sensitive group during the winter months when skin-based vitamin D production is limited in Iceland due to limited sunlight.

This study also features some limitations. Although we obtained the estimated vitamin D levels from various types of supplements available on the Icelandic market, additional details would be preferable, including the total vitamin D intake from food. However, the results of this study suggested that the contributions of dietary vitamin D sources to total vitamin D intake and status are minimal. Although the Roche method used to analyze serum 25OHD concentration in this study has been successfully applied over the years, a recent issue was identified that Roche has not yet been able to solve, in which the method occasionally falsely reports high measurements (33). Because of this issue, vitamin D results are particularly closely monitored, and all samples that report high levels are repeated for confirmation.

## Conclusion

Vitamin D supplementation is encouraged by Icelandic authorities and was widely used (approximately 80%) in the studied population. However, approximately one-third of our cohort had serum S-25OHD concentrations below adequate levels (< 50 nmol/L) during the first trimester of pregnancy, which suggests the necessary actions to increase vitamin D levels to be taken among this population. No clear association was observed between the vitamin D status and GDM in this study.

## Supplementary Material

Vitamin D status and association with gestational diabetes mellitus in a pregnant cohort in Iceland
