# Supplementary material for: Vitamin D status and association with gestational diabetes mellitus in a pregnant cohort in Iceland
Source: Food Nutr Res. 2021 Mar 23;65:10.29219/fnr.v65.5574. doi: 10.29219/fnr.v65.5574 (PMC8009083; doi:10.29219/fnr.v65.5574)
Supplement: Vitamin D status and association with gestational diabetes mellitus in a pregnant cohort in Iceland [file FNR-65-5574-s001.docx]

Supplement table 1. Number (%) of subjects taking different types of supplements containing vitamin D daily, concentration of 25OHD and number (%) of subjects defined with deficient, insufficient, and sufficient vitamin D status.

| **Cod liver oil (15–20 µg D_3_)*** | *n* (%)** | 175 (18.7) |
| --- | --- | --- |
|  | 25OHD nmol/L, mean ± SD | 66.1 ± 25.6 |
|  |  |  |
|  | 25OHD <30 nmol/L, *n* (%) | 4 (2.3) |
|  | 25OHD 30–49.9 nmol/L, *n* (%) | 39 (22.3) |
|  | 25OHD 50–74.9 nmol/L, *n* (%) | 76 (43.2) |
|  | 25OHD ≥75 nmol/L, *n* (%) | 56 (32.0) |
| **Vitamin D supplement (25–50 µg D_3_)*** | *n* (%)** | 477 (51.0) |
|  | 25OHD nmol/L, mean ± SD | 70.2 ± 25.5 |
|  |  |  |
|  | 25OHD <30 nmol/L, *n* (%) | 12 (2.5) |
|  | 25OHD 30–49.9 nmol/L, *n* (%) | 76 (15.9) |
|  | 25OHD 50–74.9 nmol/L, *n* (%) | 211 (44.2) |
|  | 25OHD ≥75 nmol/L, *n* (%) | 178 (37.3) |
| **Omega-3 with vitamin D (20 µg D_3_)*** | *n* (%)** | 164 (17.5) |
|  | 25OHD nmol/L, mean ± SD | 65.4 ± 20.3 |
|  |  |  |
|  | 25OHD <30 nmol/L, *n* (%) | 4 (2.5) |
|  | 25OHD 30–49.9 nmol/L, *n* (%) | 28 (17.1) |
|  | 25OHD 50–74.9 nmol/L, *n* (%) | 80 (48,8) |
|  | 25OHD ≥75 nmol/L, *n* (%) | 52 (31.7) |
| **Multivit with vitamin D (5–10 µg D_3_)*** | *n* (%)** | 264 (28.2) |
|  | 25OHD nmol/L, mean ± SD | 62.5 ± 23.5 |
|  |  |  |
|  | 25OHD <30 nmol/L, *n* (%) | 12 (4.5) |
|  | 25OHD 30–49.9 nmol/L, *n* (%) | 71 (26.9) |
|  | 25OHD 50–74.9 nmol/L, *n* (%) | 111 (42.0) |
|  | 25OHD ≥75 nmol/L, *n* (%) | 70 (26.5) |

*Vitamin D_3_ content of the most popular brands in each category, available in the Icelandic market.

** Some subjects reported daily use of more than one product containing vitamin D.
